# Supplementary material for: Metabolite Profiling and Transcriptome Analysis Explain the Difference in Accumulation of Bioactive Constituents in Taxilli Herba from Two Hosts
Source: Genes (Basel). 2023 May 4;14(5):1040. doi: 10.3390/genes14051040 (PMC10218634; doi:10.3390/genes14051040)
Supplement: Supplementary file 1 [file genes-14-01040-s001.zip › Supplementary Matreial of Figures.pdf]

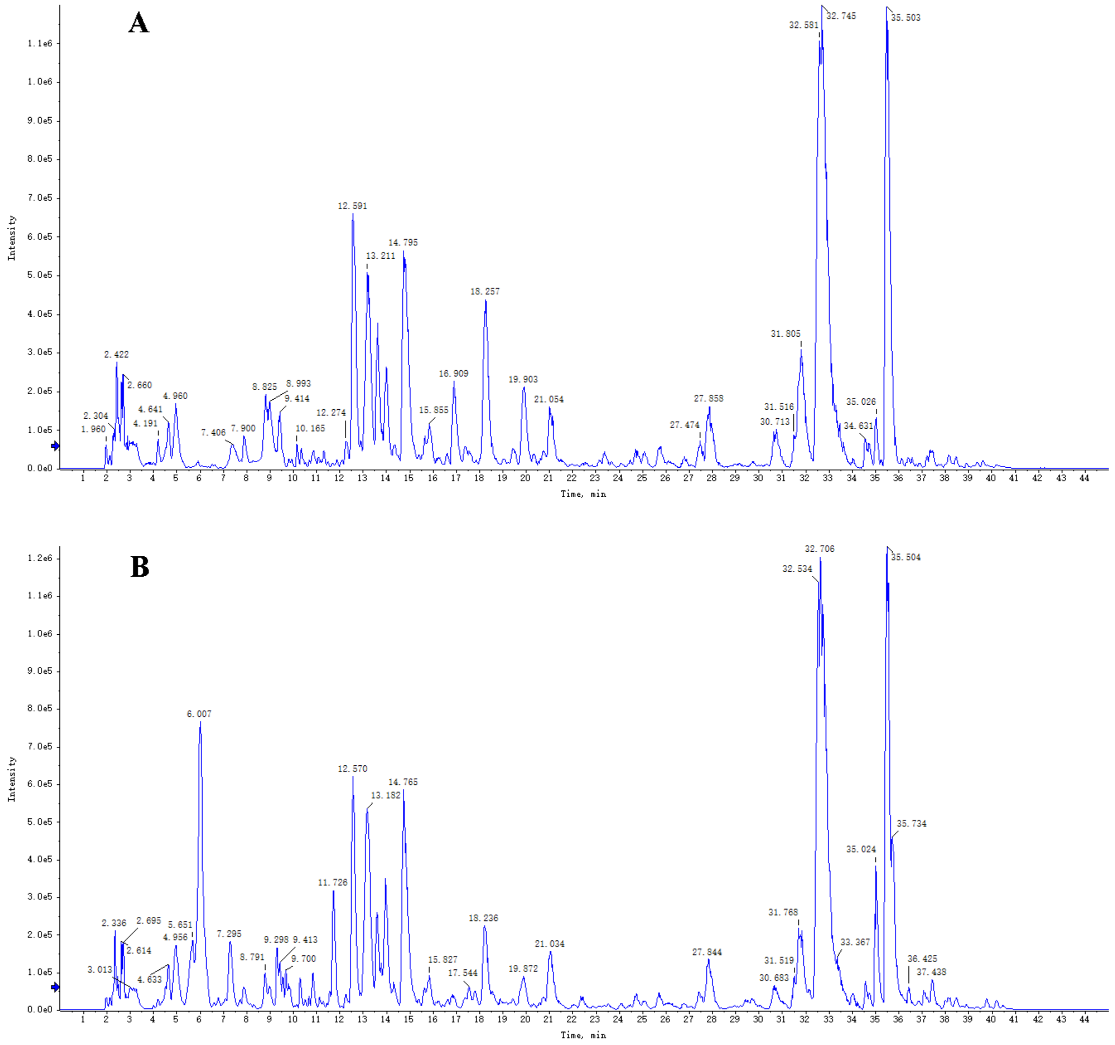

Figure S1. UFLC-Triple TOF-MS/MS base peak chromatograms of Taxilli Herba from two hosts sunder negative ion mode. (A) TH samples from *Morus alba* L. ; (B) TH samples from *Liquidambar formosana* Hance.

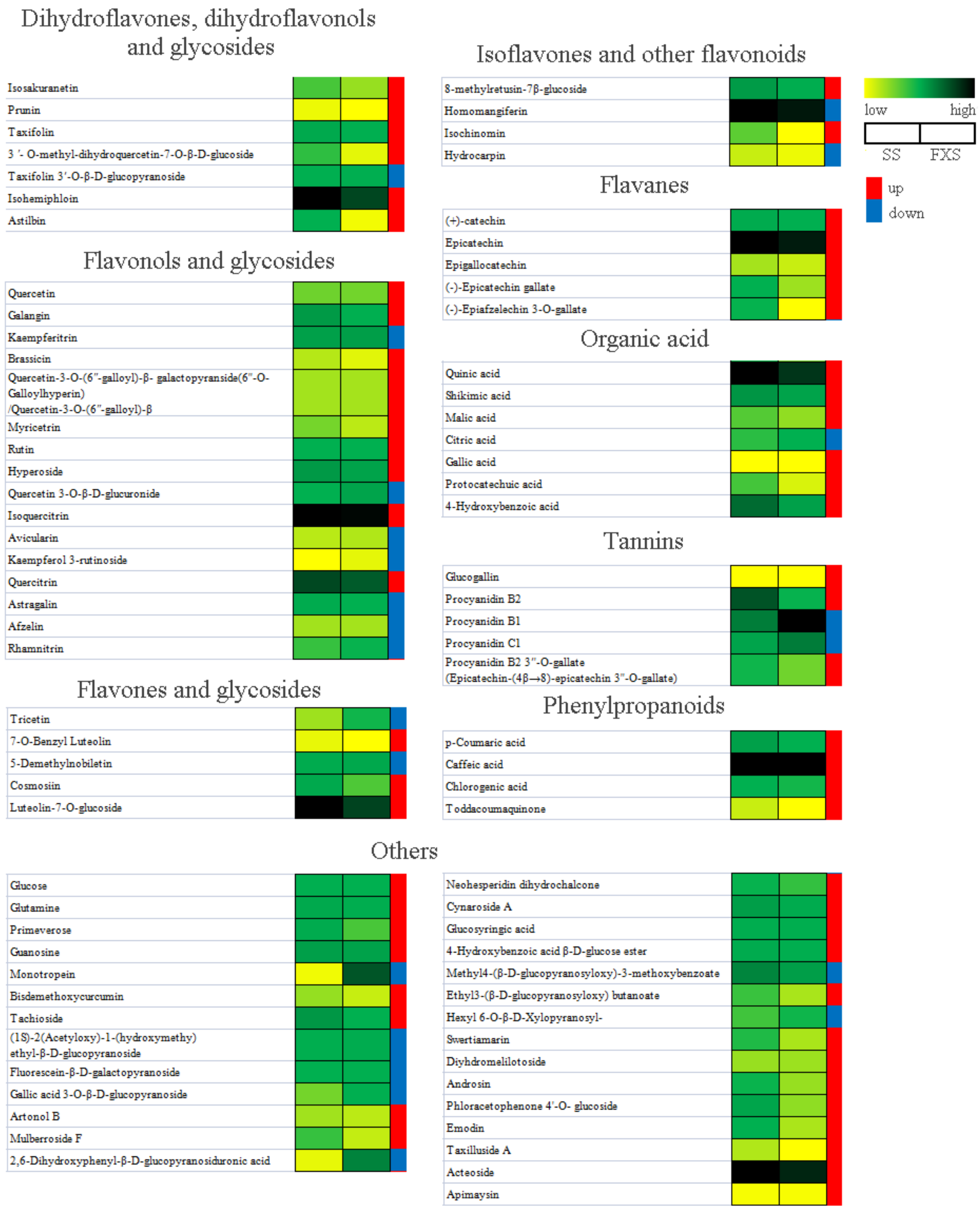

Figure S2. Heatmap of metabolite profile for 81 compounds identified in two groups of Taxilli Herba.

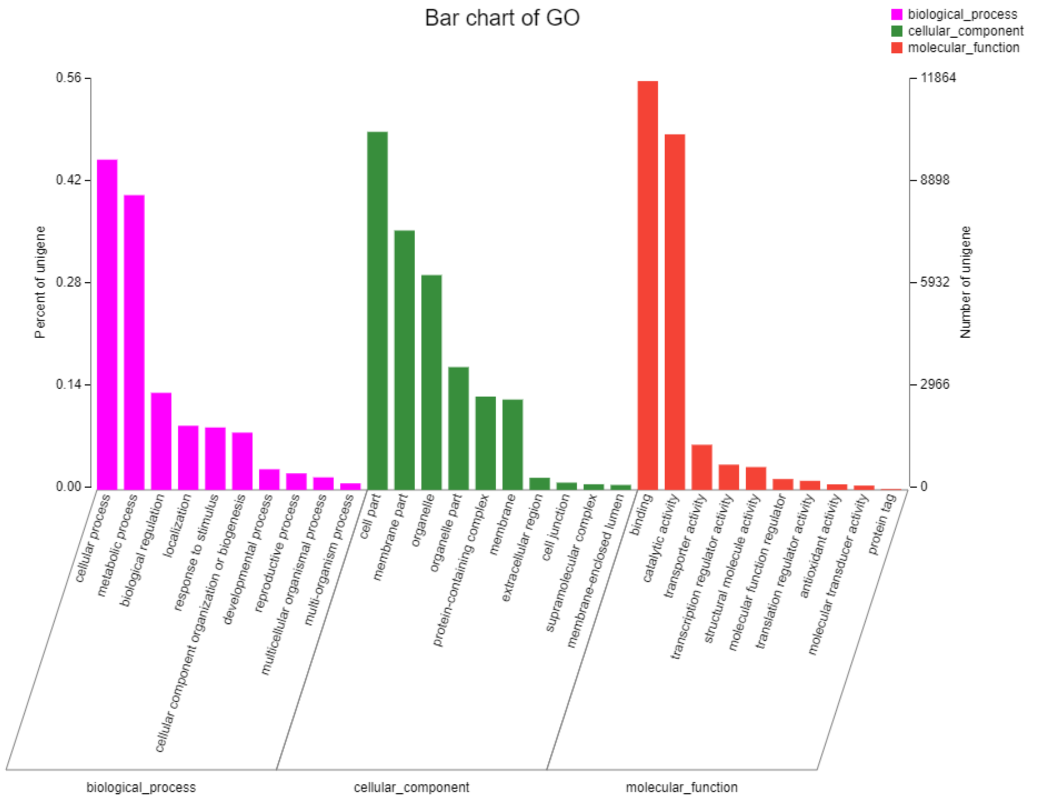

Figure S3. GO classification of all unigenes in Taxilli Herba.

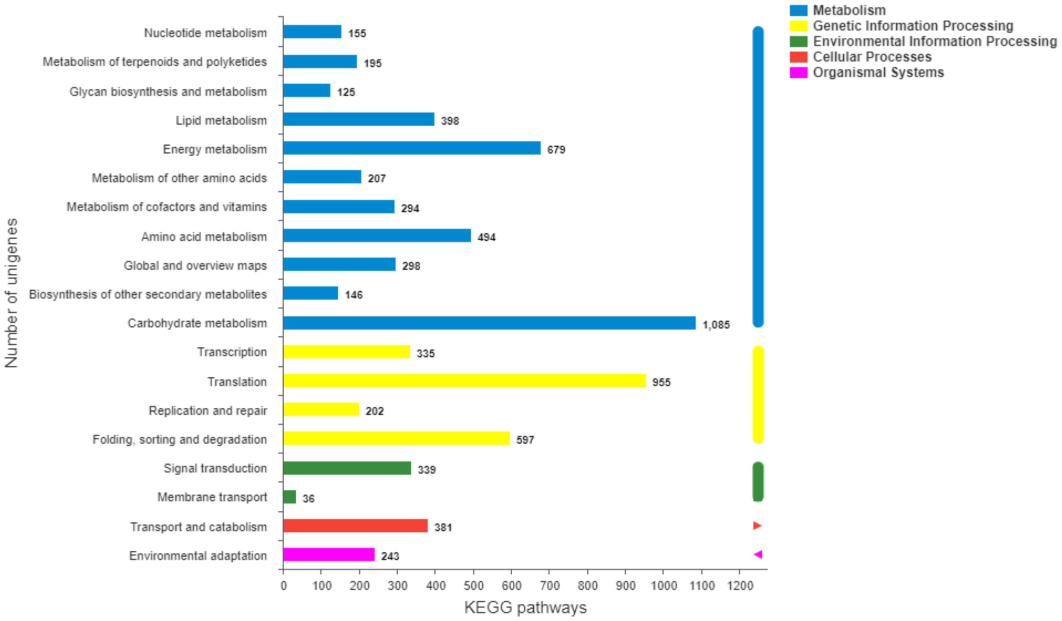

Figure S4. Pathways classification of KEGG analysis of all unigenes in Taxilli Herba.

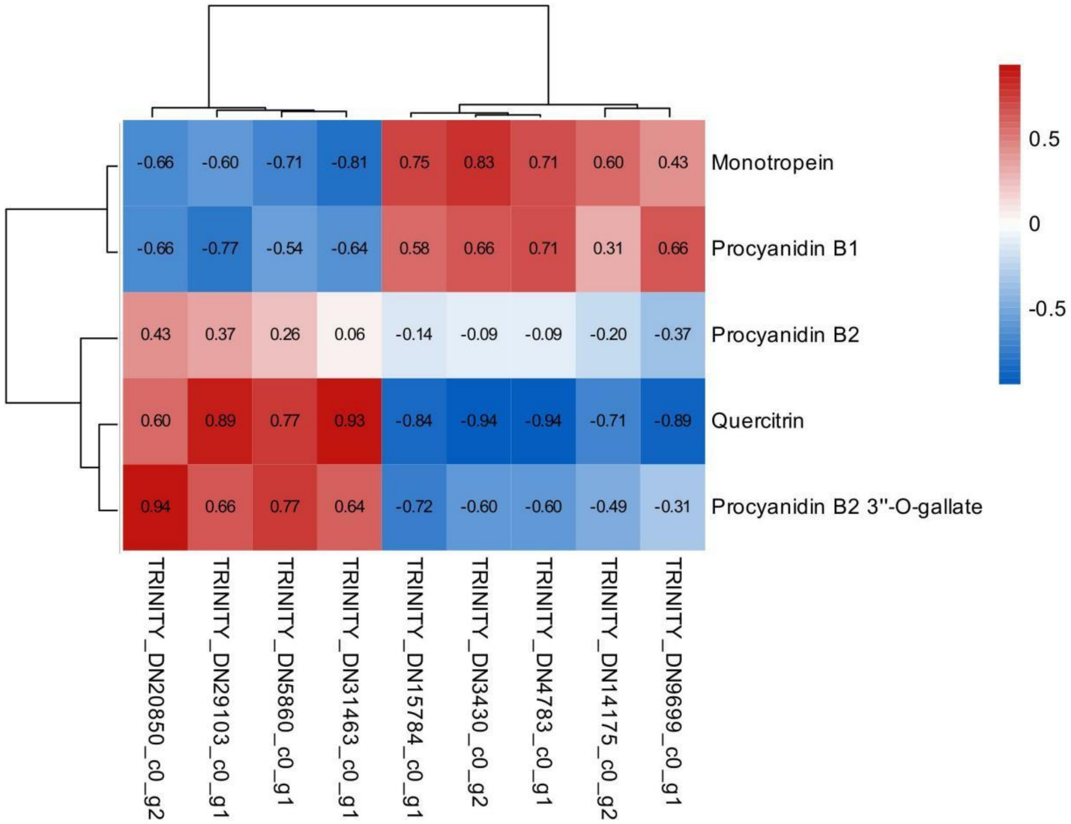

Figure S5. Correlation clustering heat map between differential components and differential genes.
